# Legislation

**Published:** 1898-01

**Authors:** 


### LEGISLATION.

### Pennsylvania.

### REGULATIONS GOVERNING THE DRIVING OR SHIPPING OF DAIRY-COWS AND NEAT-CATTLE FOR BREEDING PURPOSES INTO PENNSYLVANIA ON AND AFTER JANUARY 1, 1898.

The attention of cattle-dealers and shippers and owners is called to the following law and the rules for enforcing the same:

*An act to protect the health of the domestic animals of the Commonwealth of Pennsylvania.*

Section 1. *Be it enacted, etc.,* That the importation of dairy-cows and neat-cattle for breeding purposes into the Commonwealth of Pennsylvania is hereby prohibited, excepting when such cows and neat-cattle are accompanied by a cetificate from an inspector, whose competency and reliability are certified to by the authorities charged with the control of the diseases of domestic animals in the State from whence the cattle came, certifying that they have been examined and subjected to the tuberculin-test and are free from disease.

Sec. 2. That, in lieu of an inspection-certificate as above required, the cattle may be detained at suitable stock-yards nearest to the State line on the railroad over which they are shipped, and there examined at the expense of the owner; or cattle as above specified from points outside of the State may, under such restrictions as may be provided by the State Live-stock Sanitary Board, be shipped in quarantine to their destination in Pennsylvania, there to remain in quarantine until properly examined at the expense of the owner, and released by the State Live-stock Sanitary Board.

Sec. 3. The State Live-stock Sanitary Board is hereby authorized and empowered to prohibit the importation of domestic animals into the Commonwealth of Pennsylvania, whenever in their judgment such measures may be necessary for the proper protection of the health of the domestic animals of the Commonwealth, and to make and enforce rules and regulations governing such traffic as may from time to time be required.

Sec. 4. That any person, firm or corporate body violating the provisions of this act shall be deemed guilty of a misdemeanor, and upon conviction shall, in the proper court of the county in which such cattle are sold, offered for sale, delivered to a purchaser, or in which such cattle may be detained in transit, for each offense, forfeit and pay a fine of not less than fifty dollars or more than one hundred dollars, or be punished by imprisonment for not less than ten days, and not exceeding thirty days, either or both, at the discretion of court. Such person, firm, or corporate body shall be liable for the full amount of the damages that may result from the violation of this act.

Sec. 5. The State Live-stock Sanitary Board is hereby charged with the enforcement of this act, and is authorized to see that its provisions are obeyed, and to make, from time to time, such rules and regulations as may be necessary and proper for its enforcement.

SEC. 6. That this act shall go into effect January first, one thousand eight hundred and ninety-eight.

Approved May 26, 1897.

Rules for the enforcement of the Act of May 26, 1897:

Dairy-cows and neat-cattle for breeding purposes may be brought into Pennsylvania from other States only in accordance with one of the three following provisions:

1. The cattle may be examined and tested with tuberculin in the State from whence they come by an inspector whose competency and reliability are certified to by the authorities charged with the control of the diseases of animals in that State. Special blanks for reporting upon such examinations will be furnished by the State Live-stock Sanitary Board upon application. Cattle thus examined, found to be free from disease and brought into Pennsylvania, shall remain in the possession of the person or persons who own them when brought into Pennsylvania until the inspection-reports have been approved by a member of the State Live-stock Sanitary Board or by an agent authorized to approve such reports. After such approval the cattle can be disposed of without restriction.

2. Dairy-cows and neat-cattle for breeding purposes may, if shippers so elect, be examined and tested with tuberculin at suitable stock-yards nearest to the State line on the railroad over which they are shipped. Such examinations are to be made by inspectors approved by this board and at the expense of the owner of the cattle.

Cattle so inspected shall be marked with a suitable metal tag, or shall be accurately described, so that they can be reliably identified, and a report on the examination and test, with directions for identification, shall be submitted without delay to this board.

3. Dairy-cows and neat-cattle for breeding purposes may be brought into Pennsylvania *without previous examination only under the following conditions:*

(*a*) Notification to the State Live-stock Sanitary Board that it is proposed to bring certain dairy-cows or neat-cattle for breeding purposes into this State. Such notice must be accompanied by the number and a full and accurate description of the cattle, the names and addresses of the owner and consignee, the date upon which they are to be brought into the State, the route over which they are to be driven or shipped, and the destination.

A blank form to use in rendering this report will be sent upon application to the State Live-stock Sanitary Board.

(*b*) Such cattle shall remain in strict quarantine during transit and after they have arrived at their destination until they have been examined and tested with tuberculin by an inspector approved by this board. Under this quarantine it is required that the cattle shall be kept apart from other cattle, that they shall remain in the possession of the person or persons who bring them into this State, and that their milk shall not be sold or used without previous sterilization by boiling.

Dairy-cows or neat-cattle for breeding purposes brought into Pennsylvania under this provision that are found upon examination or test to be tuberculous, shall be strictly isolated and quarantined, their milk cannot be used for any purpose whatever without previous sterilization by boiling, and they shall not be moved to other premises excepting for slaughter. No compensation shall be allowed for such cattle.

Approved by the State Live-stock Sanitary Board at Harrisburg, Pa., November 5, 1897.

LEONARD PEARSON,
Secretary.

